# Mental Health Trajectories Among US Survivors of Adolescent and Young Adult Cancer as They Age

**DOI:** 10.1001/jamanetworkopen.2025.11430

**Published:** 2025-05-19

**Authors:** Anao Zhang, Emily Urban-Wojcik, Meghan Seewald, Bradley Zebrack

**Affiliations:** 1 University of Michigan School of Social Work, Ann Arbor; 2 University of Michigan Eisenberg Family Depression Center, Ann Arbor

## Abstract

**Importance:**

There are more than 2.1 million adult survivors of cancer diagnosed in adolescence and young adulthood (AYA) in the US. Although the mental health burden during treatment has been well documented, the long-term mental health trajectories of survivors of AYA cancer into later adulthood have not been explored.

**Objective:**

To understand the prevalence and trajectories of mental health challenges among middle-aged or older survivors of AYA cancer compared with those who received a diagnosis as adults or individuals without cancer.

**Design, Setting, and Participants:**

This cohort study used cross-sectional data from the Health and Retirement Study (HRS) to characterize mental health outcomes for US adults older than 50 years and longitudinal data to examine trajectories of mental health outcomes. The study started on September 1, 2023. HRS is a longitudinal, population-based national sample that interviews approximately 20 000 participants every 2 years since 1992 on topics related to health, employment, income, and others.

**Exposure:**

A diagnosis of cancer during AYA, defined as ages 15 to 39 years.

**Main Outcomes and Measures:**

The primary outcomes were lifetime prevalence of psychiatric issues, prescription medication for anxiety and/or depression, meeting criteria for Major Depressive Disorder using Composite International Diagnostic Interview–Short Form scoring, depression symptoms using the Center for Epidemiological Studies–Depression measure, and anxiety symptoms using items from the Beck Anxiety Inventory.

**Results:**

A total of 39 668 respondents (22 166 female [55.88%]; mean [SE] age at HRS entry, 59 [0.05] years; age range, 18-103 years; 7699 Black or African American [19.41%]; 28 459 White [71.74%]; 3402 other race [8.58%], which includes American Indian and Asian; and 108 [0.27%] missing) were identified who reported having cancer as an AYA (374 respondents), receiving a first-time cancer diagnosis after study onset as adults (5045 respondents), or never having cancer (34 249 respondents). Cross-sectional estimates revealed survivors of AYA cancer had the highest prevalence of lifetime psychiatric issues (16.36% [95% CI, 7.17%-25.55%] to 37.80% [95% CI, 26.55%-49.06%]), prescription anxiety and/or depression medication (25.10% [95% CI, 17.09%-33.10%] to 33.78% [95% CI, 23.93%-43.64%]), and meeting major depression criteria (13.13% [95% CI, 6.08%-20.18%] to 20.96% [12.91%-29.01%]) versus other cohorts. Compared with adult cancer survivors and even after adjusting for demographic covariates, AYA cancer survivors had higher odds of lifetime psychiatric issues (in 4 of 14 waves), similar odds of taking anxiety or depression medications, and higher odds of meeting major depression criteria (in 3 of 7 waves). Linear mixed-effects growth models revealed age-dependent U-shaped trajectories for depression and anxiety symptoms but significantly higher mean levels of symptoms among AYA cancer survivors. A flattening of anxiety symptoms later in life was only observed for AYA cancer survivors.

**Conclusions and Relevance:**

In this cohort study, survivors of AYA cancer reported significantly worse mental health trajectories into middle or older adulthood, compared with individuals who experienced cancer as adults or never had it. Cancer clinicians should recognize the mental health burden for this population into middle age and older adulthood.

## Key Points

**Question:**

How do mental health challenges for adult survivors of adolescent and young adult (AYA) cancer compare with those who received a diagnosis in adulthood or individuals without cancer?

**Findings:**

This cohort study used nationally representative data from the Health and Retirement Study (39 668 participants) and found that adult survivors of AYA cancer had the highest prevalence rates of mental health concerns vs those who received a diagnosis as adults or individuals without cancer. The sample as a whole followed an age-dependent U-shaped trajectory for depression and anxiety symptoms, with AYA cancer survivors reporting the highest symptom severities over the lifespan, although demonstrating a flattening of anxiety symptoms in older age.

**Meaning:**

These findings suggest that AYA cancer survivors face substantial mental health challenges into later adulthood, indicating a need for continued support.

## Teaser

This cohort study examines the prevalence and trajectories of mental health challenges among middle-aged or older survivors of adolescent and young adult cancer vs those who received a diagnosis as adults or individuals without cancer.

## Introduction

Adolescents and young adults (AYAs) are an age-specific cancer cohort (15-39 years), with 90 000 AYAs receiving new cancer diagnoses in the US annually.^1^ The AYA age includes numerous developmental milestones, such as brain maturation, relationship development, education, and career development, among others.^2,3^ Consequently, the AYA cancer cohort faces additional challenges than their counterparts from other age cohorts and people without cancer. In addition to the adverse and/or late effects common across cancer diagnoses and age cohorts, individuals with AYA cancer are disproportionately impacted by issues such as fertility or reproductive capacity, cardiotoxicity, and financial toxicity.^3,4^ Consequently, higher rates of mental health challenges have been consistently documented in the AYA cancer population, especially depression and anxiety.^5,6,7^

Existing studies have reported up to 40% of individuals with AYA cancer experience depressive symptoms, including many with major depression.^8,9^ This rate is 5 times or greater than that in the general US adult population (approximately 7.8%).^10^ Similarly, over 20% of individuals with AYA cancer have anxiety disorders, with more than 50% reporting clinically meaningful anxiety related to death.^11,12^ These much higher rates of depression and anxiety in patients with AYA cancer are likely to exacerbate their susceptibility to biopsychosocial challenges during both the active care and the posttreatment survivorship phase. However, little is known about the impacts of AYA cancer on the mental health of survivors as they age into later adulthood.

Importantly, a recent National Cancer Institute study estimated that over 2.1 million middle-aged or older adults in the US are individuals with a history of AYA cancer, a number 3 times greater than what was originally estimated (approximately 650 000).^13^ The same study also revealed that a large percentage (44%) of survivors of AYA cancer were more than 20 years from diagnosis, signifying the imperative to understand the long-term survivorship of this unique cancer cohort, especially their mental health.^14^ More importantly, studies have consistently highlighted the long-term impact of cancer and its treatment on individuals with a history of AYA cancer, such as oncofertility, financial toxicity, and cardiovascular vulnerability. These challenges further justify the importance of understanding this population’s long-term mental health trajectories.^15,16^ The purpose of this study is to understand the prevalence of mental health challenges and, more importantly, mental health trajectories of middle-aged or older adults with a history of AYA cancer. Additionally, we compare mental health prevalence and trajectories in survivors of AYA cancer with those who received a diagnosis of cancer in adulthood and individuals who never had cancer.

## Methods

This cohort study analyzed nationally representative Health and Retirement Study (HRS) data.^17^ The study was deemed exempt from the University of Michigan institutional review board, and informed consent was not needed, as data are publicly available and deidentified, in accordance with 45 CFR §46. We followed the Strengthening the Reporting of Observational Studies in Epidemiology (STROBE) reporting guidelines for cohort studies.^18^ We started the study on September 1, 2023.

### Data Source

The HRS is a nationally representative and longitudinal study of US-based adults older than 50 years and their same-household adult partners (regardless of age eligibility). Over 20 000 adults are interviewed every 2 years, dating back to 1992. The sample is replenished periodically to account for attrition, nonresponse, and maintain representativeness. The present study used data curated by the RAND Center for the Study of Aging. HRS is an ideal dataset for the current study due to its longitudinal design and nationally representative sample, including a sizable subgroup of individuals with a history of AYA cancer.

### Study Design

We determined cancer cohorts (AYA cancer, adult cancer, or no cancer) using a combination of responses to the question, “Has a doctor ever told you that you have cancer or a malignant tumor, excluding minor skin cancer?” We also determined the wave they first responded yes to this question (if ever), the date of their most recent cancer diagnosis, and their date of birth. For cancer diagnosis dates with a year but no month, the month was set to July to match how RAND computed missing months for birth dates. To compute age at diagnosis, the day of the month was set to the 15th if the diagnosis month was not imputed and set to the first if it was imputed. Time since diagnosis was computed as the difference between the interview date at each wave and the diagnosis date.

### Outcomes and Study Measures

The 3 cross-sectional mental health challenge measures were (1) reporting ever being told by a doctor they have emotional, nervous, or psychiatric problems (0 or 1; assessed all waves); (2) regularly taking medication for anxiety or depression (0 or 1; assessed at each wave 2006 and onward); and (3) meeting criteria for major depressive disorder using Composite International Diagnostic Interview–Short Form (CIDI-SF)^19^ scoring (0 or 1; assessed only during each respondent’s first wave between 1995 and 2006; assessed at each wave from 2008 onward). Respondents were screened into the CIDI-SF module if they either reported feeling depressed for 2 weeks or more in a row during the last year for at least most of the day, at least almost every day, or reported feeling a loss of interest in most things that usually gave them pleasure for 2 weeks or more in a row during the last year for at least most of the day, at least almost every day. Those meeting screening criteria for depressed affect or anhedonia were then asked about additional symptoms, including fatigue, changes in appetite, trouble falling asleep (at least nearly every night), trouble concentrating, feeling down on themselves, and having thoughts of death. Respondents who endorsed at least 5 symptoms (including depressed affect or anhedonia) were considered to have met the major depressive disorder criteria in the past year.

For longitudinal analyses examining mental health trajectories as respondents aged, we examined self-reported symptoms of depression and anxiety. Depression symptoms were measured using the Center for Epidemiological Studies–Depression (CES-D) scale^20^ at 14 waves between 1993 and 2020. Specific symptoms measured include feeling depressed, that everything is an effort, that sleep was restless, alone, sad, unable to get going, and the inverse of feeling happy and enjoying life, all or most of the time over the prior week. Symptoms were summed for a total score ranging from 0 to 8. Five anxiety symptoms from the Beck Anxiety Inventory^21^ were assessed in HRS during 6 waves between 2006 to 2020 (not measured in waves 2014 or 2016), including having a fear of the worst happening, feeling nervous, feeling hands trembling, having a fear of dying, and feeling faint over the past week on a scale of 1 to 4 (never, hardly ever, some of the time, or most of the time). The final score averaged responses across items and was set to missing if more than 2 items had missing values.

Model covariates included year of survey response (for longitudinal analyses), gender (male or female), age at interview wave (centered at 65 years and scaled by 10 years for analyses), and self-reported race (Black or African American, White, and other, which includes American Indian and Asian; these were collapsed into this category by HRS to protect respondent confidentiality). Data on race are reported here because there are documented differences in mental health prevalence rates by racial group.

### Statistical Analysis

Cross-sectional logistic regressions were conducted in 2 steps to compare prevalence estimates of each dichotomous variable at each wave of HRS as a function of wave-specific cancer cohort. Models were design-adjusted for complex survey design features and, therefore, characterized mental health outcomes for between 23 531 981 and 103 921 980 US adults older than 50 years (depending on the wave). First, wave-specific cancer cohort was entered as the sole independent variable to produce the prevalence estimates for each cancer cohort at each wave. Second, age, gender, and race were included to account for differences in demographic distributions between cancer cohorts. Postestimation Wald tests were conducted to assess whether cancer cohort as a whole had a significant association with the outcome.

Change in self-reported depression and anxiety symptoms as a function of age and wave-specific cancer cohort was modeled using mixed-effects growth models, with observations nested within respondents. The outcome was first regressed on survey years to account for historical symptom changes. The next step included age, centered on 65 years and scaled to rectify convergence issues, and a quadratic term for age. A random slope for age was included in the models to allow for individual variability in the effect of age. Next, wave-specific cancer cohort and time since cancer diagnosis were added to the model. Fourth, interactions were investigated. Finally, demographic covariates were added. Each additional step to the model was evaluated on the basis of the significance of the terms, as well as the overall model fit (eg, Akaike information criterion). Additions and interactions that were nonsignificant and/or did not improve model fit were dropped from the final model for parsimony. We used Stata/SE statistical software version 14.2 (StataCorp) to analyze the data. Statistical significance was set at 2-sided *P* < .05.

## Results

### Cohort Characteristics

Data from 42 405 respondents were accessed. We identified 39 668 eligible participants (93.5%) for this study across 15 HRS waves between 1992 and 2020, including 374 survivors of AYA cancer (aged 15-39 years at diagnosis), 5045 survivors of adult cancer (aged ≥40 years at diagnosis), and 34 249 participants who never had cancer. Respondents were excluded from analyses if they reported ever having had cancer but missing year-of-diagnosis (1008 respondents); reported their most recent cancer was diagnosed at age 40 years or older, and there was no way to determine whether they also had cancer earlier (1607 respondents); received a diagnosis when they were 14 years old or younger (8 respondents); had a data quality issue (111 respondents); or received a diagnosis as an AYA during their time as a respondent (3 respondents). Of eligible participants, 22 166 (55.88%) were female and the mean (SE) age of entry into the HRS sample was 59 (0.05) years (range, 18-103 years). Self-reported race was 7699 (19.41%) Black or African American individuals, 28 459 (71.74%) White individuals, 3402 (8.58%) individuals of other races, and 108 individuals (0.27%) with missing data.

Compared with 2737 individuals who were excluded, our final sample had a greater prevalence of men (17 502 participants [44.12%] vs 1140 participants [41.65%]; χ^2^_2_ = 6.34; *P* = .01), individuals identifying as Black or African American (7699 participants [19.45%] vs 389 participants [14.21%]) or other race (3402 participants [8.60%] vs 135 participants [4.93%]) compared with White (28 459 participants [71.94%] vs 2207 participants [80.64%]; χ^2^_2_ = 104.95; *P* < .001), and had a younger age at entry to HRS (mean [SE], 59 [0.05] vs 66 [0.22] years; *t*_3068.87_ = 29.89; *P* < .001); the excluded participants had a similar proportion of individuals with missing data on race vs the included sample (6 participants [0.22%] vs 108 participants [0.27%]). For longitudinal analyses, only participants with at least 1 wave of data for the outcome variables and complete data for full model variables were included (37 738 and for depression and 21 239 for anxiety). Sample characteristics stratified by cancer cohort are presented in Table 1. The AYA cancer cohort had a younger mean (SE) age at HRS entry (53 [0.56] years) compared with the other 2 cohorts (59 [0.06] years for no cancer and 59 [0.15] years for adult cancer) and a higher percentage of female respondents (311 participants [83.16%] for AYA cancer vs 19 399 participants [56.64%] in the no-cancer group and 2456 participants [48.68%] in the adult cancer cohort). The cohorts also had significant differences in distribution across race (where the adult cancer cohort had the highest prevalence of White-identifying individuals, the no cancer cohort had the highest prevalence of Black or African American–identifying individuals, and the AYA cancer cohort had the highest prevalence of individuals identifying as other), and education (where the AYA cancer cohort reported the greatest prevalence of individuals with some college).

**Table 1.** Descriptive Statistics by Cancer Cohort

Characteristic	Participants, No. (%)			ANOVA *F* or χ^2^ statistic	*P* value
	No cancer (n = 34 249)	Adult cancer (n = 5045)	AYA cancer (n = 374)		
Age at HRS entry, mean (SE), y	59 (0.06)	59 (0.15)	53 (0.56)	*F*_2,39 665_ = 64.48	<.001
No. of HRS waves, mean (SE)	6 (0.02)	9 (0.06)	6 (0.21)	*F*_2,39 665_ = 1019.27	<.001
Gender					
Female	19 399 (56.64)	2456 (48.68)	311 (83.16)	χ^2^_2_ = 226.92	<.001
Male	14 850 (43.36)	2589 (51.32)	63 (16.84)		
Race^a^					
Black or African American	6897 (20.20)	748 (14.86)	54 (14.44)	χ^2^_4_ = 283.76	<.001
White	24 096 (70.56)	4086 (81.17)	277 (74.06)		
Other^b^	3159 (9.25)	200 (3.97)	43 (11.50)		
Education^c^					
Less than high school	8802 (25.71)	1162 (23.03)	66 (17.65)	χ^2^_6_ = 42.82	<.001
High school graduate or General Educational Development	11 256 (32.88)	1725 (34.19)	134 (35.83)		
Some college	7669 (22.40)	1098 (21.76)	109 (29.14)		
College and above	6503 (19.00)	1060 (21.01)	65 (17.38)		

Abbreviations: ANOVA, analysis of variance; AYA, adolescent and young adult; HRS, Health and Retirement Study.

^a^ Data on race were missing for 108 respondents.

^b^ Includes American Indian and Asian, which were collapsed into this category by HRS to protect respondent confidentiality.

^c^ Data on education were missing for 19 respondents.

### Cross-Sectional Prevalence Rates

Results of the cross-sectional, design-weighted prevalence estimates and logistic regression analyses are summarized below. Table 2 presents the most recent year of pre-COVID-19 data available (2018). Full model results demonstrating the prevalence at each HRS wave are available in eTables 1 to 6 in Supplement 1. Survivors of AYA cancer had the highest prevalence rates of ever having a doctor tell them they had emotional or psychiatric issues (16.36% [95% CI, 7.17%-25.55%] to 37.80% [95% CI, 26.55%-49.06%] across waves) compared with adult cancer survivors (11.07% [95% CI, 7.44%-14.69%] to 21.18% [95% CI, 19.28%-23.08%]) and individuals without cancer (6.46% [95% CI, 5.80%-7.12%] to 22.55% [95% CI, 21.43%-23.68%]). Controlling for age, gender, and race, AYA cancer survivors had odds ratios (ORs) of 0.94 (95% CI, 0.39-2.27) to 1.85 (95% CI, 1.11-3.09) (depending on the wave) of having a lifetime prevalence of a psychiatric condition compared with an adult cancer survivor (statistically significant in 4 of 14 waves) and ORs of 1.50 (95% CI, 0.98-2.29) to 2.54 (95% CI, 1.26-5.11) compared with those without cancer (statistically significant in 13 of 15 waves). Similarly, an adult cancer survivor had ORs of 0.98 (95% CI, 0.87-1.12) to 2.59 (95% CI, 1.55-4.34) of ever having a psychiatric condition vs those without cancer (statistically significant in 9 of 14 waves).

**Table 2.** Design-Adjusted Mental Health Outcome Prevalence Estimates and ORs by Cancer Cohort in 2018

Variable	No cancer	Adult cancer	AYA cancer
Lifetime psychiatric condition (total weighted n = 99 490 438)			
Unweighted cohort, No.	14 536	1747	179
Weighted cohort, No.	88 090 975	10 317 999	1 081 464
Prevalence estimate, mean (SE), %	21.96 (0.50)	20.04 (1.11)	37.80 (5.66)
OR (95% CI)	0.89 (0.77-1.03)^a^	2.42 (1.51-3.89)^b^^,^^c^	2.16 (1.33-3.50)^c^^,^^d^
Adjusted OR (95% CI)^e^	1.01 (0.87-1.17)^a^	1.80 (1.11-2.91)^b^^,^^c^	1.81 (1.11-2.93)^c^^,^^d^
Regular anxiety or depression prescription (total weighted n = 98 818 402)			
Unweighted cohort, No.	14 428	1734	176
Weighted cohort, No.	87 517 584	10 237 039	1 063 779
Prevalence estimate, mean (SE), %	19.45 (0.53)	21.09 (1.26)	28.89 (5.84)
OR (95% CI)	1.11 (0.94-1.31)^a^	1.52 (0.87-2.67)^b^	1.68 (0.95-2.98)^d^
Adjusted OR (95% CI)^e^	1.22 (1.04-1.43)^a^^,^^c^	1.12 (0.63-2.00)^b^	1.37 (0.77-2.42)^d^
Meets major depressive episode criteria in past year (total weighted n = 96 395 385)^f^			
Unweighted cohort, No.	14 007	1636	177
Weighted cohort, No.	85 462 064	9 866 657	1 066 664
Prevalence estimate, mean (SE), %	7.46 (0.33)	5.76 (0.72)	20.22 (5.14)
OR (95% CI)	0.76 (0.57-1.00)^a^	4.15 (2.11-8.15)^b^^,^^c^	3.14 (1.65-5.98)^c^^,^^d^
Adjusted OR (95% CI)^e^	1.01 (0.75-1.35)^a^	2.49 (1.29-4.82)^b^^,^^c^	2.51 (1.34-4.70)^c^^,^^d^

Abbreviations: AYA, adolescent and young adult; OR, odds ratio.

^a^ Data are for adult vs no cancer.

^b^ Data are for AYA vs adult cancer.

^c^ Significant at *P* < .05.

^d^ Data are for AYA vs no cancer.

^e^ Adjusted for age, gender, and race.

^f^ Major depressive episode is defined using the Composite International Diagnostic Interview–Short Form scale.

Across the 8 waves with data on prescription medication, AYA cancer survivors had the highest prevalence of taking prescription depression and anxiety medications (25.10% [95% CI, 17.09%-33.10%] to 33.78% [95% CI, 23.93%-43.64%]), followed by adult cancer survivors (16.77% [95% CI, 14.13%-19.41%] to 21.38% [95% CI, 19.02%-23.74%]) and individuals without cancer (16.09% [95% CI, 15.29%-16.90%] to 19.45% [95% CI, 18.40%-20.49%]). ORs comparing odds between AYA and adult cancer survivors were nonsignificant when adjusting for age, gender, and race (ranging from 0.96 [95% CI, 0.60-1.56] to 1.68 [95% CI, 0.98-2.86]). Adjusting for covariates, an AYA cancer survivor had ORs of 1.29 (95% CI, 0.84-1.96) to 2.00 (95% CI, 1.26-3.17) for regularly taking depression and anxiety prescription medication compared with those without cancer (statistically significant in 4 of 8 waves). Adult cancer survivors had ORs of 1.12 (95% CI, 0.92-1.38) to 1.34 (95% CI, 1.14-1.57) for taking a prescription depression and anxiety medication compared with those who had never had cancer (statistically significant in 6 of 8 waves), adjusting for age, gender, and sex.

In the 7 waves of HRS where respondents were universally screened for major depression using CIDI-SF, AYA cancer survivors had the highest prevalence rates for meeting major depression criteria in the previous year, with prevalence rates from 13.13% (95% CI, 6.08%-20.18%) to 20.96% (95% CI, 12.91%-29.01%) compared with adult cancer survivors (6.06% [95% CI, 4.68%-7.44%] to 7.23% [95% CI, 5.57%-8.90%]) and individuals without cancer (6.51% [95% CI, 5.96%-7.06%] to 8.45% [95% CI, 7.86%-9.04%]). The ORs of an AYA cancer survivor having a depressive episode in the prior year were 1.18 (95% CI, 0.58-2.39) to 2.49 (95% CI, 1.29-4.82) greater than an adult cancer survivor of the same age, gender, and racial group (statistically significant in 3 of 7 waves) and 1.61 (95% CI, 0.88-2.96) to 2.51 (95% CI, 1.34-4.70) greater than an individual without cancer (statistically significant in 4 of 7 waves). Adult cancer survivors had similar odds of having a depressive episode compared with individuals who had never had cancer when adjusting for age and gender in all but 1 wave (ORs ranging from 1.01 [95% CI, 0.75-1.35] to 1.37 [95% CI, 1.06-1.76]).

### Growth Models

**Figure 1.**
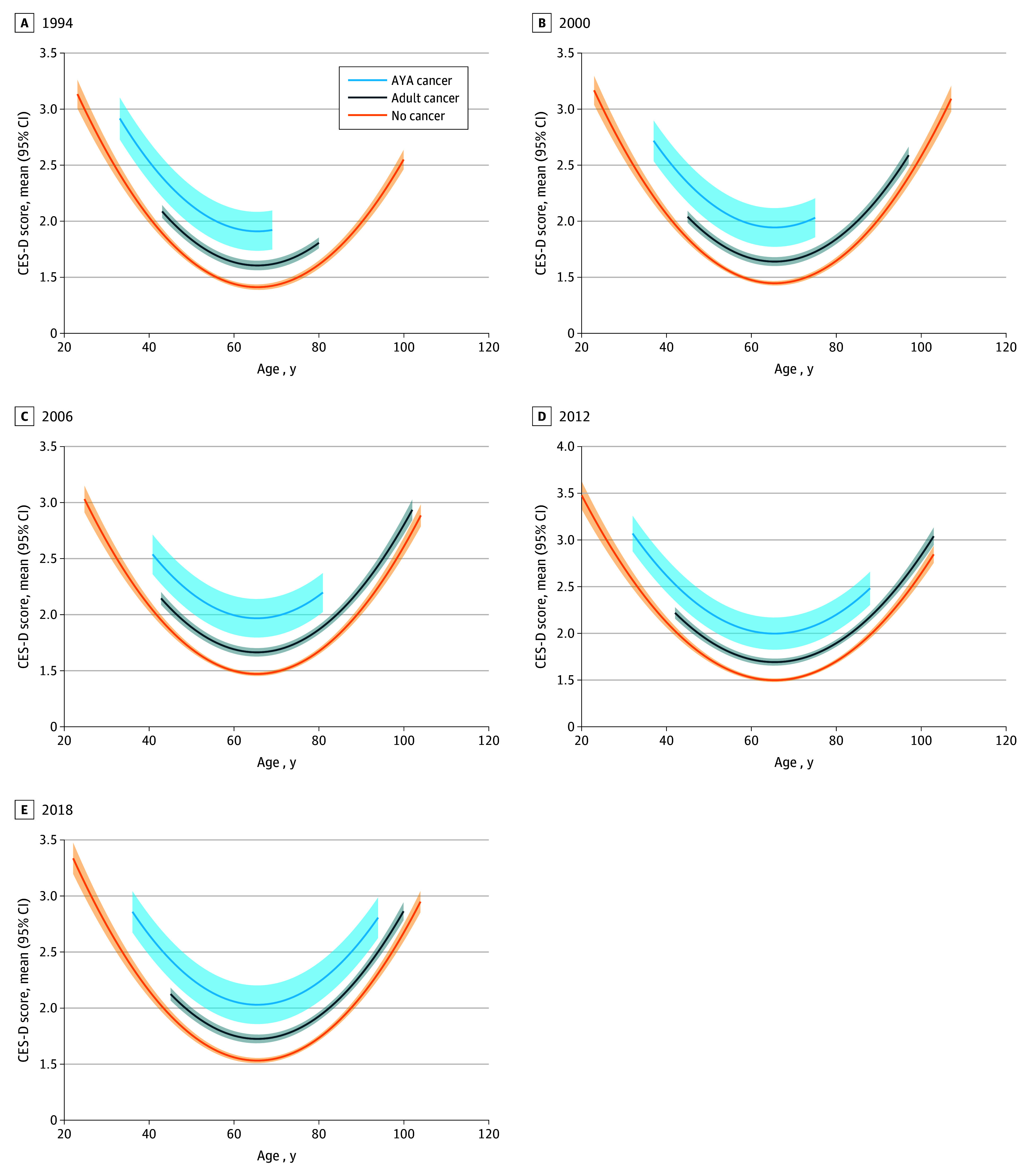
Estimated Center for Epidemiological Studies–Depression (CES-D) Scores by Age and Cancer Cohort at Select Health and Retirement Survey Years Solid lines denote mean scores, and shaded areas denote 95% CIs. AYA indicates adolescent and young adult.

Growth models revealed that CES-D scores increased each year of the study for the whole sample on average. Furthermore, model building revealed a significant U-shaped quadratic association between age and CES-D score, resulting in both age as a random effect and age-squared being included in the model. Next, the fixed-effect of wave-specific cancer cohort was added to the model, revealing significant between-cohort mean differences in CES-D trajectories, where the AYA cancer cohort had the highest mean levels of CES-D score, followed by the adult cancer cohort (Figure 1). Adding an age-by-cancer cohort interaction did not reveal any significant changes, suggesting that all 3 cohorts followed similar U-shaped trajectories (the final model is shown in Table 3). Covariates of gender, race, and time since diagnosis did not change the nature of the results.

**Table 3.** Mixed-Effects Growth Models of Depression and Anxiety Symptoms

Variable^a^	Coefficient (95% CI)				
	Model 1	Model 2	Model 3	Model 4	Model 5
CES-D depressive symptoms					
Intercept	1.51 (1.49 to 1.54)^b^	1.41 (1.39 to 1.44)^b^	1.91 (1.73 to 2.08)^b^	1.89 (1.71 to 2.08)^b^	2.16 (1.94 to 2.39)^b^
Year of survey	0.008 (0.007 to 0.010)^b^	0.006 (0.005 to 0.007)^b^	0.005 (0.004 to 0.006)^b^	0.005 (0.004 to 0.006)^b^	0.002 (0.00 to 0.003)^c^
Age	NA	−0.002 (−0.014 to 0.011)	−0.009 (−0.022 to 0.004)	−0.032 (−0.152 to 0.088)	0.025 (0.012 to 0.038^b^
Age squared	NA	0.096 (0.090 to 0.103)^b^	0.095 (0.089 to 0.101)^b^	0.096 (0.090 to 0.103)^b^	0.093 (0.087 to 0.099)^b^
Cancer cohort: no cancer	NA	NA	−0.50 (−0.67 to −0.32)^b^	−0.49 (−0.67 to −0.30)^b^	−0.67 (−0.89 to −0.44)^b^
Cancer cohort: adult cancer	NA	NA	−0.30 (−0.48 to −0.13)^d^	−0.27 (−0.46 to −0.09)^d^	−0.42 (−0.64 to −0.21)^b^
Age by no cancer	NA	NA	NA	0.02 (−0.10 to 0.14)	NA
Age by adult cancer	NA	NA	NA	0.00 (−0.13 to 0.12)	NA
Years since diagnosis	NA	NA	NA	NA	−0.08 (−0.01 to −0.003)^d^
Male gender	NA	NA	NA	NA	−0.38 (−0.41 to −0.34)^b^
Race: Black or African American	NA	NA	NA	NA	0.43 (0.39 to 0.47)^b^
Race: other	NA	NA	NA	NA	0.40 (0.44 to 0.57)^b^
AIC	893 574.90	893 300.40	893 155.90	893 157.20	892 092.10
Likelihood ratio test	NA	278.50	148.53	2.71	1071.87
*P* value	NA	<.001	<.001	.26	<.001
Mean of BAI symptoms					
Intercept	1.58 (1.57 to 1.59)^b^	1.56 (1.55 to 1.57)^b^	1.69 (1.61 to 1.77)^b^	1.68 (1.60 to 1.75)^b^	1.70 (1.56 to 1.84)^b^
Year of survey	0.002 (0.001 to 0.003)^b^	0.002 (0.001 to 0.003)^b^	0.002 (0.001 to 0.003)^b^	0.002 (0.001 to 0.003)^b^	0.001 (−0.000 to .002)
Age	NA	−0.020 (−0.027 to −0.013)^b^	−0.022 (−0.030 to −0.015)^b^	−0.109 (−0.176 to −0.043)^d^	−0.089 (−0.163 to −0.016)^c^
Age squared	NA	0.024 (0.020 to 0.029)^b^	0.024 (0.020 to 0.029)^b^	0.025 (0.021 to 0.029)^b^	0.023 (0.019 to 0.028)^b^
Cancer cohort: no cancer	NA	NA	−0.14 (−0.22 to −0.06)^b^	−0.13 (−0.20 to −0.05)^d^	−0.14 (−0.28 to −0.001)^c^
Cancer cohort: adult cancer	NA	NA	−0.09 (−0.17 to −0.01)^c^	−0.06 (−0.14 to 0.02)	−0.07 (−0.20 to 0.06)
Age by no cancer	NA	NA	NA	0.09 (0.02 to 0.16)^d^	0.08 (0.01 to 0.15)^c^
Age by adult cancer	NA	NA	NA	0.07 (0.00 to 0.14)	0.06 (−0.01 to 0.14)
Years since diagnosis	NA	NA	NA	NA	0.00 (−0.004 to 0.002)
Male gender	NA	NA	NA	NA	−0.08 (−0.09 to −0.06)^b^
Race: Black or African American	NA	NA	NA	NA	0.14 (0.12 to 0.16)^b^
Race: other	NA	NA	NA	NA	0.13 (0.10 to 0.16)^b^
AIC	62 846.84	62 688.77	62 660.21	62 654.32	62 320.00
Likelihood ratio test	NA	162.07	32.56	9.89	342.32
*P* value	NA	<.001	<.001	.007	<.001

Abbreviations: AIC, Akaike information criterion; BAI, Beck Anxiety Inventory; CES-D, Center for Epidemiological Studies–Depression; NA, not applicable.

^a^ Data for CES-D are based on 37 738 respondents and 235 254 observations across Health and Retirement Survey (HRS) waves starting in 1993 and beyond. Data for BAI are based on 21 239 respondents and 38 150 observations across 6 HRS waves between 2006 and 2020 (not included in waves 2014 or 2016). The intraclass correlation coefficient was 53% for CES-D and 57% for BAI. Outcomes represent the sum of CES-D symptoms present for a possible range of 0 to 8 (top) and a mean of 5 anxiety symptoms from the BAI, for a possible range of 1 to 4. The year of survey variable was centered on 1993 for CES-D and 2006 for BAI. The age variable was centered at 65 years and scaled by a factor of 10 years. The adolescent and young adult cancer group acted as the cancer group reference. White acted as the race group reference. Other race includes American Indian and Asian, which were collapsed into this category by HRS to protect respondent confidentiality. Each likelihood ratio test compares with the previous model, except the CES-D model 5, which compares with model 3.

^b^ *P* < .001.

^c^ *P* < .05.

^d^ *P* < .01.

**Figure 2.**
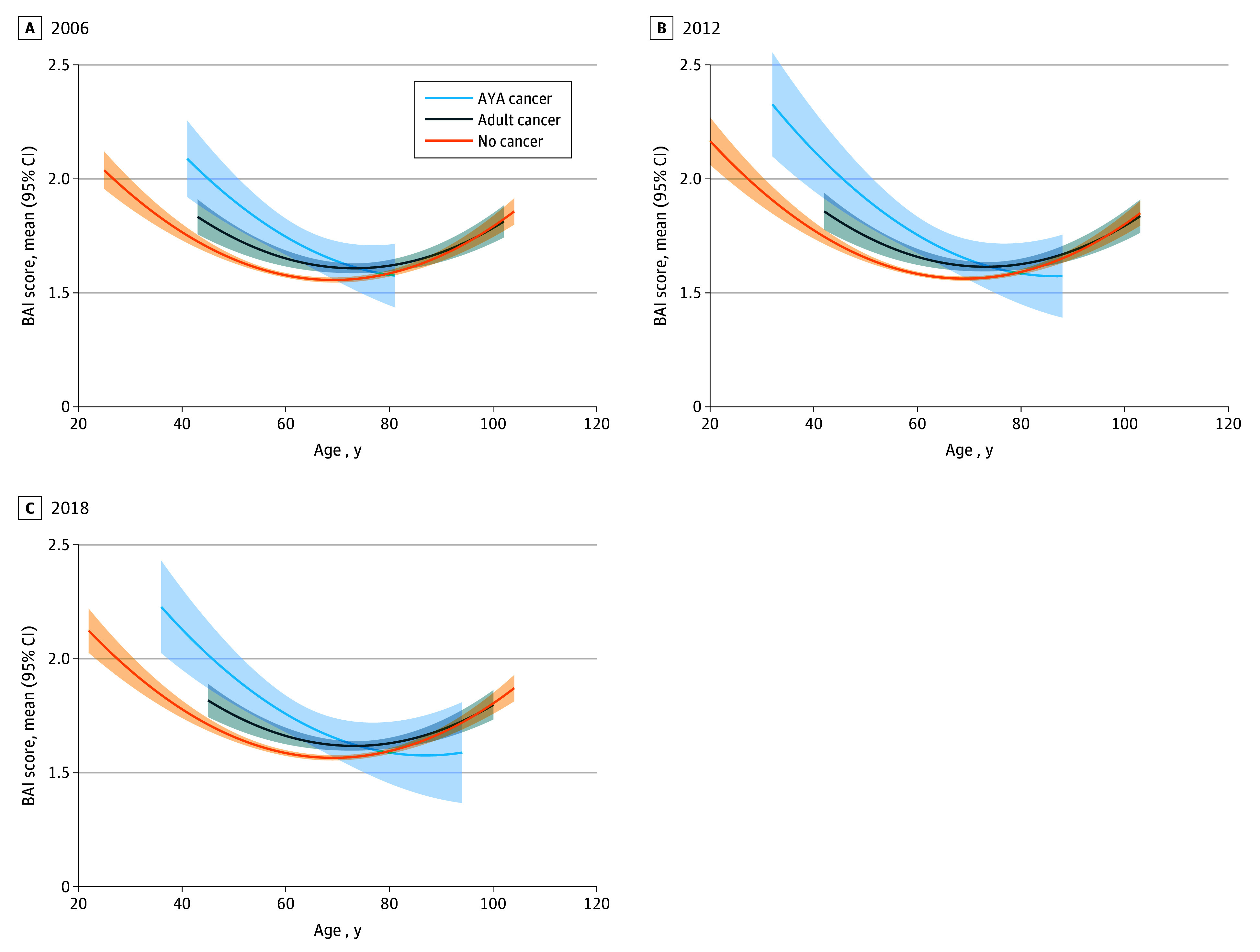
Estimated Beck Anxiety Inventory (BAI) Average Scores by Age and Cancer Cohort at Select Health and Retirement Survey Years Solid lines denote mean scores, and shaded areas denote 95% CIs. AYA indicates adolescent and young adult.

A second set of mixed-effect growth models were fit for symptoms of anxiety (Table 3), which increased each year of the study. Age and quadratic age terms illustrated a U-shaped association where anxiety symptoms were the highest at younger and older ages and lowest near middle age. Fixed effects of wave-specific cancer cohort demonstrated AYA cancer survivors had the highest mean level of anxiety symptoms followed by the adult cancer cohort. A significant age-by-cancer cohort interaction revealed a U-shaped association for no-cancer and adult cancer cohorts but a flattening trend for AYA cancer survivors (Figure 2), although the difference in curvature between the 2 cancer cohorts was not significant. The addition of covariates did not change the nature of the findings.

## Discussion

The long-term mental health effects of cancer and its treatment on individuals with a history of AYA cancer are compellingly demonstrated in this cohort study. Adults who received a diagnosis of and were treated for cancer between ages 15 to 39 years reported significantly greater prevalence and severity of depression and anxiety than did older adult cancer survivors or those without cancer. With over 2.1 million middle-aged or older adult survivors of AYA cancer in the US, this age-distinct cancer cohort will continue to grow rapidly with advancements in therapeutics that improve survival rates.^13,14^ As individuals with a history of AYA cancer live longer, this study attends to a key knowledge gap in understanding this cohort’s long-term mental health trajectories.^22^ Consistent with what is well-established about the unique challenges confronting this cohort, findings revealed the highest prevalence rate of lifetime psychiatric issues, prescribed psychiatric medications, and depressive episodes in individuals with a history of AYA cancer. Notably, the revealed nationally representative estimates of depression prevalence rate in adult survivors of AYA cancer (13.13%-20.96%) were much higher than those reported in previous studies (ie, <9%)^23,24^ and align with existing studies documenting substantially elevated risk of mental disorders in patients with AYA cancer during and immediately following cancer treatment.^8,9,25^ Additionally, for all 3 cross-sectional outcomes, female participants had higher prevalence rates than male participants across HRS waves, persisting across cancer cohorts. This may be associated with higher proportions of AYA-specific breast cancers.

Similar to existing studies portraying the longitudinal trend of depression across the lifespan,^26,27,28^ the mixed growth model revealed a U-shaped depression trajectory for all cohorts as they age. Notably, survivors of AYA cancer consistently displayed the greatest severity of depression across all age periods, substantiating the prolonged and disproportionate impact of cancer-related adverse and/or late effects confronting the AYA cancer survivor cohort years into their posttreatment survivorship phase.^29,30^ Clinicians are encouraged to be aware of the vulnerability to depression among individuals with a history of AYA cancer while also integrating the U-shaped nature of the depression trajectory into patient education and depression prevention.

Interestingly, unlike the general and adult cancer populations that exhibited a U-shaped anxiety trajectory, individuals with a history of AYA cancer demonstrated a flattening of symptoms over time, especially into older adulthood (ages >75 years and older), with anxiety symptom scores converging with or falling below those of adult cancer survivors and those without cancer. Such a pattern may reflect survival bias, where only the healthiest survivors of AYA cancer live to older ages, potentially reducing this cohort’s reported anxiety.^31,32^ Additionally, there have been studies suggesting that older cancer survivors may adapt more effectively over time and find ways to better manage long-term psychological effects,^33,34^ or they might experience less-acute or health-related anxiety due to shifts in life priorities and perspectives with advancing age.^35^

### Limitations

This study has limitations that should be mentioned. First, although the study leverages a large, nationally representative longitudinal dataset, the reliance on self-reported data may introduce recall bias, particularly for historical cancer diagnoses. Second, HRS does not capture important detailed cancer treatment and surveillance information, which could influence mental health outcomes. Third, we had to exclude over 2000 participants for whom we could not precisely determine their cancer cohorts. Such an exclusion, however, does not impact the findings’ generalizability as we accounted for complex survey design. Fourth, given the possibility of confounding by unknown factors, it is likely that we did not account for all important factors.

## Conclusions

This cohort study is among the first that highlights the sustained mental health challenges confronting survivors of AYA cancer, revealing this cohort’s significantly higher level of mental health trajectories (ie, depression and anxiety). Growth curve models revealed that although the full sample exhibited a U-shaped trajectory of depression symptoms across the lifespan, AYA cancer survivors consistently reported higher levels of depressive symptoms, indicating an enduring impact of cancer-related stressors. Interestingly, anxiety symptoms among AYA survivors flattened over time, suggesting possible resilience. As the number of AYA cancer survivors continues to grow, it remains a critical priority to improve survivorship outcomes using a holistic approach.

## Notes

Supplement 1. **eTable 1.** Weighted prevalence rates of ever reporting psychiatric problems at each HRS wave by cancer group **eTable 2.** Weighted prevalence rates of ever reporting psychiatric problems at each HRS wave by cancer group adjusted for age, gender, and race **eTable 3.** Weighted prevalence rates of regularly taking prescription medication for anxiety or depression at each HRS wave by cancer group **eTable 4.** Weighted prevalence rates of regularly taking prescription medication for anxiety or depression at each HRS wave by cancer group adjusted for age, gender, and race **eTable 5.** Weighted prevalence rates of meeting major depression criteria in past year at each HRS wave by cancer group **eTable 6.** Weighted prevalence rates of meeting major depression criteria in past year at each HRS wave by cancer group adjusted for age, gender, and race

Supplement 2.
Data Sharing Statement
