# Supplementary material for: Mental Health Trajectories Among US Survivors of Adolescent and Young Adult Cancer as They Age
Source: JAMA Netw Open. 2025 May 19;8(5):e2511430. doi: 10.1001/jamanetworkopen.2025.11430 (PMC12090028; doi:10.1001/jamanetworkopen.2025.11430)
Supplement: Supplement 1. — eTable 1. Weighted prevalence rates of ever reporting psychiatric problems at each HRS wave by cancer group eTable 2. Weighted prevalence rates of ever reporting psychiatric problems at each HRS wave by cancer group adjusted for age, gender, and race eTable 3. Weighted prevalence rates of regularly taking prescription medication for anxiety or depression at each HRS wave by cancer group eTable 4. Weighted prevalence rates of regularly taking prescription medication for anxiety or depression at each HRS wave by cancer group adjusted for age, gender, and race eTable 5. Weighted prevalence rates of meeting major depression criteria in past year at each HRS wave by cancer group eTable 6. Weighted prevalence rates of meeting major depression criteria in past year at each HRS wave by cancer group adjusted for age, gender, and race [file jamanetwopen-e2511430-s001.pdf]

Supplemental Online Content

Zhang A, Urban-Wojcik E, Seewald M, Zebrack B. Mental health trajectories among US survivors of adolescent and young adult cancer as they age. *JAMA Netw Open*. 2025;8(5):e2511430. doi:10.1001/jamanetworkopen.2025.11430

eTable 1. Weighted prevalence rates of ever reporting psychiatric problems at each HRS wave by cancer group
eTable 2. Weighted prevalence rates of ever reporting psychiatric problems at each HRS wave by cancer group adjusted for age, gender, and race
eTable 3. Weighted prevalence rates of regularly taking prescription medication for anxiety or depression at each HRS wave by cancer group
eTable 4. Weighted prevalence rates of regularly taking prescription medication for anxiety or depression at each HRS wave by cancer group adjusted for age, gender, and race
eTable 5. Weighted prevalence rates of meeting major depression criteria in past year at each HRS wave by cancer group
eTable 6. Weighted prevalence rates of meeting major depression criteria in past year at each HRS wave by cancer group adjusted for age, gender, and race

This supplemental material has been provided by the authors to give readers additional information about their work.

© 2025 Zhang A et al. *JAMA Network Open*.

eTable 1. Weighted prevalence rates of ever reporting psychiatric problems at each HRS wave by cancer group

		No cancer		Adult Cancer		AYA Cancer		
Year	Total Weighted *N*	Weighted Group *n*	Prevalence % (95% CI)	Weighted Group *n*	Prevalence % (95% CI)	Weighted Group *n*	Prevalence % (95% CI)	**Post-estimation Wald test for cancer group**
1992	23,531,981	23,323,926	6.46^{a} (5.80, 7.12)	--	--	208,055	16.36^{b} (7.17, 25.55)	$F(1, 52) = 8.87, p = .004$
1993/1994	42,255,965	41,791,437	7.14^{a} (6.75, 7.52)	258,543	17.14^{b} (9.54, 24.73)	205,985	18.94^{b} (9.01, 28.88)	$F(2, 51) = 14.27, p < .001$
1995/1996	39,321,817	38,102,813	8.09^{a} (7.56, 8.63)	1,022,246	11.07^{a} (7.44, 14.69)	196,758	21.68^{b} (10.57, 32.79)	$F(2, 51) = 7.97, p = .001$
1998	63,863,703	61,434,479	9.23^{a} (8.67, 9.79)	1,949,486	11.66^{ab} (8.96, 14.37)	479,738	20.66^{b} (11.41, 29.92)	$F(2, 51) = 8.56, p < .001$
2000	60,569,326	57,236,005	10.05^{a} (9.48, 10.62)	2,857,162	12.47^{a} (9.84, 15.10)	476,159	23.56^{b} (13.72, 33.41)	$F(2, 51) = 9.73, p < .001$
2002	58,565,039	54,130,518	11.76^{a} (11.13, 12.40)	3,973,324	13.03^{a} (10.66, 15.40)	461,197	26.87^{b} (17.13, 36.62)	$F(2, 51) = 8.91, p = .001$
2004	76,501,114	71,035,465	12.91^{a} (12.25, 13.57)	4,868,615	13.16^{a} (11.02, 15.29)	597,034	26.88^{b} (18.19, 35.56)	$F(2, 55) = 8.33, p = .001$
2006	73,219,209	66,923,885	14.92^{a} (14.22, 15.62)	5,725,160	14.60^{a} (12.46, 16.73)	570,164	30.29^{b} (20.22, 40.36)	$F(2, 55) = 7.33, p = .002$
2008	69,491,737	62,540,686	16.03^{a} (15.18, 16.88)	6,439,132	16.87^{a} (14.41, 19.33)	511,919	32.27^{b} (22.04, 42.50)	$F(2, 55) = 8.70, p = .001$
2010	90,389,915	82,594,697	16.41^{a} (15.61, 17.21)	6,991,416	18.18^{a} (15.97, 20.38)	803,802	26.57^{b} (19.22, 33.92)	$F(2, 55) = 6.20, p = .004$
2012	86,713,283	77,932,473	17.99^{a} (17.15, 18.83)	8,046,130	18.32^{a} (16.54, 20.10)	734,680	29.13^{b} (20.89, 37.38)	$F(2, 55) = 5.44, p = .007$
2014	82,851,151	73,529,819	19.12^{a} (18.27, 19.97)	8,614,550	18.99^{a} (17.08, 20.91)	706,782	31.13^{b} (21.08, 41.19)	$F(2, 55) = 3.76, p = .030$
2016	103,921,980	93,314,184	20.22^{a} (19.27, 21.16)	9,420,084	21.18^{a} (19.28, 23.08)	1,187,712	31.38^{b} (22.48, 40.28)	$F(2, 79) = 3.92, p = .024$
2018	99,490,438	88,090,975	21.96^{a} (20.96, 22.97)	10,317,999	20.04^{a} (17.82, 22.26)	1,081,464	37.80^{b} (26.55, 49.06)	$F(2, 79) = 7.69, p = .001$
2020	94,680,724	82,763,801	22.55^{a} (21.43, 23.68)	10,891,132	20.53^{a} (18.41, 22.66)	1,025,791	35.00^{b} (24.52, 45.45)	$F(2, 79) = 5.92, p = .004$

Note. Due to differences in early HRS data collection procedures, RAND combined 1993/1994 and 1995/1996 based on entry cohort. Given the definition of the Adult cancer group as developing cancer after entry into HRS, these cells are null for the first wave. Margins with the same superscripts within HRS year do not differ significantly from one another at $p < .05$.

Odds Ratios (95% Confidence Interval) for cancer group contrasts

Year	**Adult vs. No**	**AYA vs. No**	**AYA vs. Adult**
1992	--	**2.83 (1.40, 5.71)**	--
1993/1994	**2.69 (1.59, 4.54)**	**3.04 (1.58, 5.86)**	1.13 (0.47, 2.74)
1995/1996	1.41 (0.98, 2.03)	**3.14 (1.61, 6.14)**	**2.22 (1.02, 4.85)**
1998	1.30 (0.99, 1.70)	**2.56 (1.43, 4.58)**	1.97 (1.00, 3.91)
2000	1.27 (1.00, 1.63)	**2.76 (1.59, 4.78)**	**2.16 (1.16, 4.05)**
2002	1.12 (0.90, 1.40)	**2.76 (1.67, 4.56)**	**2.45 (1.40, 4.31)**
2004	1.02 (0.85, 1.23)	**2.48 (1.59, 3.87)**	**2.43 (1.48, 3.97)**
2006	0.97 (0.83, 1.15)	**2.48 (1.55, 3.97)**	**2.54 (1.51, 4.27)**
2008	1.06 (0.89, 1.26)	**2.50 (1.58, 3.93)**	**2.35 (1.42, 3.89)**
2010	1.13 (0.97, 1.32)	**1.84 (1.28, 2.65)**	**1.63 (1.12, 2.36)**
2012	1.02 (0.90, 1.16)	**1.87 (1.27, 2.77)**	**1.83 (1.20, 2.81)**
2014	0.99 (0.87, 1.14)	**1.91 (1.20, 3.06)**	**1.93 (1.18, 3.15)**
2016	1.06 (0.93, 1.20)	**1.80 (1.18, 2.75)**	**1.70 (1.12, 2.58)**
2018	0.89 (0.77, 1.03)	**2.16 (1.33, 3.50)**	**2.42 (1.51, 3.89)**
2020	0.88 (0.78, 1.01)	**1.85 (1.16, 2.94)**	**2.08 (1.31, 3.32)**

Bold = significant OR at $p < .05$

eTable 2. Weighted prevalence rates of ever reporting psychiatric problems at each HRS wave by cancer group adjusted for age, gender, and race

		No cancer		Adult Cancer		AYA Cancer		
Year	Total Weighted *N*	Weighted Group *n*	Prevalence % (95% CI)	Weighted Group *n*	Prevalence % (95% CI)	Weighted Group *n*	Prevalence % (95% CI)	**Post-estimation Wald test for cancer group**
1992	23,531,981	23,323,926	6.47^{a} (5.81, 7.13)	--	--	208,055	14.08^{b} (6.05, 22.11)	$F(1, 52) = 6.21, p = .016$
1993/1994	42,238,016	41,773,488	7.15^{a} (6.76, 7.54)	258,543	16.56^{b} (9.41, 23.71)	205,985	15.68^{b} (6.95, 24.40)	$F(2, 51) = 11.03, p < .001$
1995/1996	39,310,684	38,091,680	8.10^{a} (7.56, 8.63)	1,022,246	11.53^{b} (7.88, 15.18)	196,758	18.13^{b} (8.05, 28.21)	$F(2, 51) = 6.52, p = .003$
1998	63,837,287	61,412,408	9.22^{a} (8.67, 9.77)	1,945,141	12.78^{b} (9.75, 15.80)	479,738	17.25^{b} (9.63, 24.87)	$F(2, 51) = 8.36, p < .001$
2000	60,554,098	57,225,240	10.04^{a} (9.47, 10.60)	2,852,699	13.51^{b} (10.64, 16.37)	476,159	19.14^{b} (10.86, 27.43)	$F(2, 51) = 8.52, p < .001$
2002	58,551,849	54,117,328	11.72^{a} (11.09, 12.35)	3,973,324	14.16^{b} (11.66, 16.65)	461,197	22.21^{b} (13.66, 30.77)	$F(2, 51) = 7.37, p = .002$
2004	76,487,356	71,021,707	12.85^{a} (12.21, 13.50)	4,868,615	14.38^{a} (12.05, 16.71)	597,034	23.34^{b} (15.57, 31.12)	$F(2, 55) = 6.79, p = .002$
2006	73,211,342	66,916,018	14.82^{a} (14.15, 15.50)	5,725,160	16.11^{a} (13.71, 18.51)	570,164	26.03^{b} (17.21, 34.86)	$F(2, 55) = 5.99, p = .004$
2008	69,483,451	62,532,400	15.89^{a} (15.08, 16.70)	6,439,132	18.71^{b} (15.98, 21.45)	511,919	27.66^{c} (18.60, 36.72)	$F(2, 55) = 8.53, p < .001$
2010	90,253,919	82,458,701	16.32^{a} (15.54, 17.11)	6,991,416	20.11^{b} (17.47, 22.74)	803,802	23.37^{b} (16.63, 30.11)	$F(2, 55) = 6.93, p = .002$
2012	86,582,089	77,801,279	17.88^{a} (17.04, 18.71)	8,046,130	20.08^{b} (18.00, 22.16)	734,680	25.90^{b} (18.34, 33.47)	$F(2, 55) = 5.90, p = .005$
2014	82,710,172	73,391,022	18.97^{a} (18.12, 19.82)	8,612,368	20.77^{ab} (18.51, 23.02)	706,782	27.47^{b} (18.21, 36.72)	$F(2, 55) = 3.43, p = .040$
2016	103,647,702	93,044,360	20.12^{a} (19.19, 21.05)	9,415,630	22.82^{b} (20.67, 24.98)	1,187,712	27.23^{ab} (19.16, 35.31)	$F(2, 79) = 4.21, p = .018$
2018	99,214,901	87,827,454	21.77^{a} (20.79, 22.76)	10,305,983	21.88^{a} (19.45, 24.31)	1,081,464	33.20^{b} (22.82, 43.58)	$F(2, 79) = 2.98, p = .057$
2020	94,428,656	82,542,290	22.40 (21.30, 23.49)	10,860,575	22.12 (19.86, 24.38)	1,025,791	30.10 (20.59, 39.61)	$F(2, 79) = 1.54, p = .222$

Note. Due to differences in early HRS data collection procedures, RAND combined 1993/1994 and 1995/1996 based on entry cohort. Given the definition of the Adult cancer group as developing cancer after entry into HRS, these cells are null for the first wave. Margins with the same superscripts within HRS year do not differ significantly from one another at $p \leq .05$.

Odds Ratios (95% Confidence Interval) for cancer group contrasts adjusted for age, gender, and race

Year	**Adult vs. No**	**AYA vs. No**	**AYA vs. Adult**
1992	--	**2.38 (1.18, 4.80)**	--
1993/1994	**2.59 (1.55, 4.34)**	**2.43 (1.23, 4.78)**	0.94 (0.39, 2.27)
1995/1996	**1.48 (1.04, 2.12)**	**2.54 (1.26, 5.11)**	1.71 (0.76, 3.86)
1998	**1.45 (1.09, 1.92)**	**2.07 (1.18, 3.62)**	1.43 (0.73, 2.80)
2000	**1.41 (1.09, 1.81)**	**2.15 (1.24, 3.72)**	1.53 (0.81, 2.87)
2002	**1.25 (1.00, 1.54)**	**2.18 (1.30, 3.64)**	1.75 (0.98, 3.11)
2004	1.14 (0.94, 1.38)	**2.08 (1.34, 3.24)**	**1.83 (1.11, 3.01)**
2006	1.10 (0.93, 1.31)	**2.04 (1.29, 3.24)**	**1.85 (1.11, 3.09)**
2008	**1.22 (1.02, 1.46)**	**2.05 (1.31, 3.21)**	**1.68 (1.01, 2.78)**
2010	**1.29 (1.09, 1.53)**	**1.57 (1.09, 2.28)**	1.22 (0.82, 1.80)
2012	**1.16 (1.01, 1.33)**	**1.62 (1.09, 2.39)**	1.40 (0.91, 2.16)
2014	1.12 (0.96, 1.31)	**1.63 (1.02, 2.62)**	1.45 (0.88, 2.41)
2016	**1.18 (1.03, 1.35)**	1.50 (0.98, 2.29)	1.27 (0.83, 1.96)
2018	1.01 (0.87, 1.17)	**1.81 (1.11, 2.93)**	**1.80 (1.11, 2.91)**
2020	0.98 (0.87, 1.12)	1.51 (0.94, 2.41)	1.53 (0.94, 2.48)

Bold = significant OR at $p < .05$

© 2025 Zhang A et al. *JAMA Network Open.*

eTable 3. Weighted prevalence rates of regularly taking prescription medication for anxiety or depression at each HRS wave by cancer group

		No cancer		Adult Cancer		AYA Cancer		
Year	Total Weighted *N*	Weighted Group *n*	Prevalence % (95% CI)	Weighted Group *n*	Prevalence % (95% CI)	Weighted Group *n*	Prevalence % (95% CI)	**Post-estimation Wald test for cancer group**
2006	73,028,395	66,747,226	16.09[a] (15.29, 16.90)	5,711,005	17.12[a] (14.92, 19.31)	570,164	32.07[b] (22.15, 41.99)	$F(2, 55) = 9.13$ $p < .001$
2008	69,327,791	62,410,265	16.35[a] (15.63, 17.08)	6,405,607	16.77[a] (14.13, 19.41)	511,919	31.03[b] (21.22, 40.85)	$F(2, 55) = 6.89$ $p = .002$
2010	89,131,400	81,383,131	17.48[a] (16.74, 18.21)	6,961,712	18.31[a] (15.73, 20.88)	786,557	27.15[b] (20.25, 34.05)	$F(2, 55) = 4.84$ $p = .012$
2012	86,368,843	77,605,238	17.85[a] (17.04, 18.67)	8,028,925	20.39[b] (17.68, 23.09)	734,680	25.10[b] (17.09, 33.10)	$F(2, 55) = 5.74$ $p = .006$
2014	82,544,092	73,255,434	18.50[a] (17.56, 19.44)	8,584,461	21.00[b] (18.62, 23.37)	704,197	28.29[b] (18.32, 38.26)	$F(2, 55) = 4.96$, $p = .011$
2016	103,308,631	92,763,552	18.45[a] (17.60, 19.31)	9,369,057	21.38[b] (19.02, 23.74)	1,176,022	33.78[c] (23.93, 43.64)	$F(2, 79) = 8.50$ $p = .001$
2018	98,818,402	87,517,584	19.45[a] (18.40, 20.49)	10,237,039	21.09[a] (18.58, 23.59)	1,063,779	28.89[a] (17.26, 40.52)	$F(2, 79) = 1.99$ $p = .144$
2020	93,765,857	81,968,772	19.15[a] (17.99, 20.31)	10,795,113	21.32[ab] (18.66, 23.98)	1,001,972	29.58[b] (19.38, 39.77)	$F(2, 79) = 3.76$ $p = .028$

Note. Margins with the same superscripts within HRS year do not differ significantly from one another at $p < .05$.

Odds Ratios (95% Confidence Interval) for cancer group contrasts

Year	Adult vs. No	AYA vs. No	AYA vs. Adult
2006	1.08 (0.91, 1.27)	**2.46 (1.56, 3.89)**	**2.29 (1.36, 3.83)**
2008	1.03 (0.85, 1.26)	**2.30 (1.46, 3.62)**	**2.23 (1.34, 3.74)**
2010	1.06 (0.89, 1.26)	**1.76 (1.23, 2.53)**	**1.66 (1.17, 2.37)**
2012	**1.18 (1.00, 1.38)**	**1.54 (1.02, 2.33)**	1.31 (0.81, 2.12)
2014	**1.17 (1.02, 1.34)**	**1.74 (1.07, 2.82)**	1.48 (0.91, 2.43)
2016	**1.20 (1.04, 1.39)**	**2.25 (1.45, 3.50)**	**1.88 (1.20, 2.92)**
2018	1.11 (0.94, 1.31)	1.68 (0.95, 2.98)	1.52 (0.87, 2.67)
2018	1.14 (0.98, 1.34)	**1.77 (1.10, 2.85)**	1.55 (0.96, 2.50)

Bold = significant OR at $p < .05$

eTable 4. Weighted prevalence rates of regularly taking prescription medication for anxiety or depression at each HRS wave by cancer group adjusted for age, gender, and race

		No cancer		Adult Cancer		AYA Cancer		
Year	Total Weighted *N*	Weighted Group *n*	Prevalence % (95% CI)	Weighted Group *n*	Prevalence % (95% CI)	Weighted Group *n*	Prevalence % (95% CI)	**Post-estimation Wald test for cancer group**
2006	73,020,528	66,739,359	16.01[a] (15.24, 16.78)	5,711,005	18.44[b] (16.03, 20.85)	570,164	27.25[b] (18.33, 36.18)	$F(2, 55) = 8.71$, $p < .001$
2008	69,319,505	62,401,979	16.26[a] (15.57, 16.96)	6,405,607	17.89[ab] (15.06, 20.72)	511,919	26.17[b] (17.23, 35.11)	$F(2, 55) = 4.67$, $p = .013$
2010	88,996,977	81,248,708	17.39[a] (16.65, 18.12)	6,961,712	20.00[ab] (17.06, 22.94)	786,557	23.33[b] (17.19, 29.47)	$F(2, 55) = 3.16$, $p = .065$
2012	86,237,649	77,474,044	17.73[a] (16.93, 18.53)	8,028,925	22.23[b] (19.46, 25.01)	734,680	21.61[ab] (14.45, 28.77)	$F(2, 55) = 8.50$, $p < .001$
2014	82,403,113	73,116,637	18.38[a] (17.46, 19.30)	8,582,279	22.62[b] (20.09, 25.15)	704,197	24.05[ab] (15.23, 32.87)	$F(2, 55) = 7.89$, $p = .001$
2016	103,034,353	92,493,728	18.39[a] (17.55, 19.24)	9,364,603	22.82[b] (20.24, 25.41)	1,176,022	28.78[b] (20.08, 37.48)	$F(2, 79) = 9.25$, $p < .001$
2018	98,548,622	87,259,820	19.35[a] (18.34, 20.36)	10,225,023	22.56[b] (20.00, 25.12)	1,063,779	24.52[ab] (14.41, 34.63)	$F(2, 79) = 3.45$, $p = .037$
2020	93,513,789	81,747,261	19.07[a] (17.95, 20.19)	10,764,556	22.62[b] (19.94, 25.30)	1,001,972	24.63[ab] (15.66, 33.59)	$F(2, 79) = 4.79$, $p = .012$

Note. Margins with the same superscripts within HRS year do not differ significantly from one another at $p < .05$.

Odds Ratios (95% Confidence Interval) for cancer group contrasts adjusted for age, gender, and race

Year	Adult vs. No	AYA vs. No	AYA vs. Adult
2006	**1.19 (1.01, 1.41)**	**2.00 (1.26, 3.17)**	1.68 (0.98, 2.86)
2008	1.12 (0.92, 1.38)	**1.85 (1.16, 2.97)**	1.65 (0.96, 2.84)
2010	1.19 (0.99, 1.44)	**1.46 (1.01, 2.10)**	1.22 (0.86, 1.74)
2012	**1.34 (1.14, 1.57)**	1.29 (0.84, 1.96)	0.96 (0.60, 1.56)
2014	**1.31 (1.13, 1.51)**	1.42 (0.87, 2.30)	1.09 (0.66, 1.79)
2016	**1.32 (1.13, 1.55)**	**1.83 (1.18, 2.84)**	1.38 (0.88, 2.18)
2018	**1.22 (1.04, 1.43)**	1.37 (.77, 2.42)	1.12 (.63, 2.00)
2020	**1.25 (1.07, 1.46)**	1.40 (.86, 2.27)	1.12 (.65, 1.85)

Bold = significant OR at $p < .05$

© 2025 Zhang A et al. *JAMA Network Open.*

eTable 5. Weighted prevalence rates of meeting major depression criteria in past year at each HRS wave by cancer group

		**No cancer**		**Adult Cancer**		**AYA Cancer**		
Year	Total Weighted *N*	Weighted Group *n*	Prevalence % (95% CI)	Weighted Group *n*	Prevalence % (95% CI)	Weighted Group *n*	Prevalence % (95% CI)	**Post-estimation Wald test for cancer group**
2008	66,324,418	59,757,518	6.51[a] (5.96, 7.06)	6,066,508	6.74[a] (5.03, 8.44)	500,392	13.85[b] (5.43, 22.27)	$F(2, 55) = 2.78$, $p = .071$
2010	86,388,430	79,132,442	8.45[a] (7.86, 9.04)	6,462,975	6.06[b] (4.68, 7.44)	793,013	18.93[c] (11.89, 25.96)	$F(2, 55) = 10.59$, $p < .001$
2012	83,331,301	75,024,112	7.31[a] (6.67, 7.95)	7,577,656	7.23[ab] (5.57, 8.90)	729,533	13.13[b] (6.08, 20.18)	$F(2, 55) = 2.20$, $p = .121$
2014	79,477,662	70,620,647	7.55[a] (6.93, 8.17)	8,153,766	6.50[a] (5.00, 8.00)	703,249	15.82[b] (8.81, 22.84)	$F(2, 55) = 5.16$, $p = .009$
2016	100,412,940	90,264,949	8.19[a] (7.56, 8.81)	8,970,353	6.95[a] (5.50, 8.39)	1,177,638	20.96[b] (12.91, 29.01)	$F(2, 79) = 10.17$, $p < .001$
2018	96,395,385	85,462,064	7.46[a] (6.81, 8.12)	9,866,657	5.76[a] (4.33, 7.19)	1,066,664	20.22[b] (9.99, 30.44)	$F(2, 79) = 8.87$, $p < .001$
2020	90,973,668	79,704,593	7.62[a] (6.92, 8.32)	10,247,067	6.83[a] (5.23, 8.43)	1,022,008	15.25[b] (7.90, 22.61)	$F(2, 79) = 4.13$, $p = .020$

Note. Margins with the same superscripts within HRS year do not differ significantly from one another at $p < .05$.

Odds Ratios (95% Confidence Interval) for cancer group contrasts

Year	**Adult vs. No**	**AYA vs. No**	**AYA vs. Adult**
2008	1.04 (0.78, 1.39)	**2.31 (1.13, 4.70)**	**2.22 (1.03, 4.81)**
2010	**0.70 (0.54, 0.91)**	**2.53 (1.61, 3.97)**	**3.62 (2.08, 6.30)**
2012	0.99 (0.78, 1.25)	**1.92 (1.02, 3.58)**	1.94 (0.95, 3.96)
2014	0.85 (0.66, 1.10)	**2.30 (1.35, 3.92)**	**2.70 (1.45, 5.04)**
2016	0.84 (0.67, 1.04)	**2.97 (1.79, 4.94)**	**3.55 (2.04, 6.20)**
2018	0.76 (0.57, 1.00)	**3.14 (1.65, 5.98)**	**4.15 (2.11, 8.15)**
2020	0.89 (0.67, 1.17)	**2.18 (1.23, 3.88)**	**2.46 (0.85, 1.48)**

Bold = significant OR at $p < .05$

eTable 6. Weighted prevalence rates of meeting major depression criteria in past year at each HRS wave by cancer group adjusted for age, gender, and race

		**No cancer**		**Adult Cancer**		**AYA Cancer**		
Year	Total Weighted *N*	Weighted Group *n*	Prevalence % (95% CI)	Weighted Group *n*	Prevalence % (95% CI)	Weighted Group *n*	Prevalence % (95% CI)	**Post-estimation Wald test for cancer group**
2008	66,316,132	59,749,232	6.40[a] (5.89, 6.91)	6,066,508	8.37[a] (6.24, 10.50)	500,392	11.07[a] (4.15, 17.98)	$F(2, 55) = 3.39$, $p = .041$
2010	86,263,477	79,007,489	8.24[a] (7.68, 8.80)	6,462,975	8.80[a] (6.94, 10.66)	793,013	16.07[b] (10.09, 22.06)	$F(2, 55) = 6.19$, $p = .004$
2012	83,200,709	74,893,520	7.15[a] (6.57, 7.74)	7,577,656	9.48[b] (7.20, 11.75)	729,533	10.95[ab] (5.23, 16.68)	$F(2, 55) = 5.17$, $p = .009$
2014	79,340,675	70,485,842	7.39[a] (6.82, 7.97)	8,151,584	8.14[ab] (6.22, 10.06)	703,249	13.18[b] (7.14, 19.23)	$F(2, 55) = 3.47$, $p = .038$
2016	100,143,538	89,999,195	7.98[a] (7.37, 8.59)	8,966,705	9.48[a] (7.37, 11.58)	1,177,638	16.48[b] (10.12, 22.84)	$F(2, 79) = 7.07$, $p = .002$
2018	96,121,602	85,200,297	7.28[a] (6.65, 7.92)	9,854,641	7.33[a] (5.45, 9.20)	1,066,664	16.22[b] (8.06, 24.39)	$F(2, 79) = 4.27$, $p = .017$
2020	90,726,137	79,483,983	7.45[a] (6.77, 8.12)	10,220,146	8.63[a] (6.51, 10.75)	1,022,008	12.20[a] (6.25, 18.15)	$F(2, 79) = 2.50$, $p = .089$

Note. Margins with the same superscripts within HRS year do not differ significantly from one another at $p < .05$.

Odds Ratios (95% Confidence Interval) for cancer group contrasts adjusted for age, gender, and race

Year	**Adult vs. No**	**AYA vs. No**	**AYA vs. Adult**
2008	1.34 (0.99, 1.81)	1.84 (0.89, 3.78)	1.37 (0.62, 3.02)
2010	1.08 (0.84, 1.38)	**2.17 (1.38, 3.42)**	**2.02 (1.17, 3.48)**
2012	**1.37 (1.06, 1.76)**	1.61 (0.88, 2.96)	1.18 (0.58, 2.39)
2014	1.11 (0.85, 1.45)	**1.93 (1.12, 3.32)**	1.73 (0.69, 1.17)
2016	1.21 (0.95, 1.55)	**2.33 (1.41, 3.84)**	**1.92 (1.09, 3.39)**
2018	1.01 (0.75, 1.35)	**2.51 (1.34, 4.70)**	**2.49 (1.29, 4.82)**
2020	1.18 (0.87, 1.59)	1.75 (0.99, 3.10)	1.49 (0.77, 2.85)

Bold = significant OR at $p < .05$

© 2025 Zhang A et al. *JAMA Network Open.*
