# Supplementary material for: Mental Health Trajectories Among US Survivors of Adolescent and Young Adult Cancer as They Age
Source: JAMA Netw Open. 2025 May 19;8(5):e2511430. doi: 10.1001/jamanetworkopen.2025.11430 (PMC12090028; doi:10.1001/jamanetworkopen.2025.11430)
Supplement: Supplement 2. — Data Sharing Statement [file jamanetwopen-e2511430-s002.pdf]

Data Sharing Statement

Zhang. Mental Health Trajectories Among US Survivors of Adolescent and Young Adult Cancer as They Age. *JAMA Netw Open*. Published May 19, 2025.
doi:10.1001/jamanetworkopen.2025.11430

Data
Data available: Yes
Data types: Deidentified participant data
How to access data: https://hrs.isr.umich.edu/about
When available: With publication

Supporting Documents
Document types: None

Additional Information
Who can access the data: Anyone requesting the data
Types of analyses: Analytical codes, with reasonable request
Mechanisms of data availability: Publically available dataset
